# The Persister Character of Clinical Isolates of *Staphylococcus aureus* Contributes to Faster Evolution to Resistance and Higher Survival in THP-1 Monocytes: A Study With Moxifloxacin

**DOI:** 10.3389/fmicb.2020.587364

**Published:** 2020-11-23

**Authors:** Tiep K. Nguyen, Frédéric Peyrusson, Magali Dodémont, Nhung H. Pham, Hoang A. Nguyen, Paul M. Tulkens, Françoise Van Bambeke

**Affiliations:** ^1^Pharmacologie Cellulaire et Moléculaire, Louvain Drug Research Institute, Université catholique de Louvain (UCLouvain), Brussels, Belgium; ^2^Department of Pharmaceutical Industry, Hanoi University of Pharmacy, Hanoi, Vietnam; ^3^Centre National de Référence des Staphylocoques, Laboratoire Hospitalier Universitaire de Bruxelles (LHUB-ULB) Site Anderlecht, Hôpital Erasme – Cliniques Universitaires de Bruxelles, Brussels, Belgium; ^4^Department of Microbiology, Bach Mai Hospital, Hanoi, Vietnam; ^5^Microbiology Department, Hanoi Medical University, Hanoi, Vietnam; ^6^The National Center for Drug Information and Adverse Drug Reactions Monitoring, Hanoi University of Pharmacy, Hanoi, Vietnam

**Keywords:** *Staphylococcus aureus*, persister, intracellular infection, resistance, intracellular survival, moxifloxacin

## Abstract

*Staphylococcus aureus* may cause relapsing infections. We previously showed that *S. aureus* SH1000 surviving intracellularly to bactericidal antibiotics are persisters. Here, we used 54 non-duplicate clinical isolates to assess links between persistence, resistance evolution, and intracellular survival, using moxifloxacin throughout as test bactericidal antibiotic. The relative persister fraction (RPF: percentage of inoculum surviving to 100× MIC moxifloxacin in stationary phase culture for each isolate relative to ATCC 25923) was determined to categorize isolates with low (≤10) or high (>10) RPF. Evolution to resistance (moxifloxacin MIC ≥ 0.5 mg/L) was triggered by serial passages at 0.5× MIC (with daily concentration readjustments). Intracellular moxifloxacin maximal efficacy (E_max_) was determined by 24 h concentration-response experiments [pharmacodynamic model (Hill-Langmuir)] with infected THP-1 monocytes exposed to moxifloxacin (0.01 to 100× MIC) after phagocytosis. Division of intracellular survivors was followed by green fluorescence protein dilution (FACS). Most (30/36) moxifloxacin-susceptible isolates showed low RPF but all moxifloxacin-resistant (*n* = 18) isolates harbored high RPF. Evolution to resistance of susceptible isolates was faster for those with high vs. low RPF (with SOS response and topoisomerase-encoding genes overexpression). Intracellularly, moxifloxacin E_max_ was decreased (less negative) for isolates with high vs. low RPF, independently from resistance. Moxifloxacin intracellular survivors were non-dividing. The data demonstrate and quantitate persisters in clinical isolates of *S. aureus*, and show that this phenotype accelerates resistance evolution and is associated with intracellular survival in spite of high antibiotic concentrations. Isolates with high RPF may represent a possible cause of treatment failure not directly related to resistance in patients receiving active antibiotics.

## Introduction

Persisters, defined as subpopulations of bacteria that do not multiply but still survive in the presence of a bactericidal antibiotic, are suspected of being a potential cause of therapeutic failure in spite of the use of active antibiotics (Van den Bergh et al., 2017; Jung et al., 2019). This phenotype is not genetically inherited and is fully reversible upon antibiotic removal (Cohen et al., 2013), thereby escaping detection through conventional antibiotic susceptibility testing. Persisters grow normally in conventional media, and need, therefore, to be distinguished from small colony variants (SCVs), which are phenotypic subpopulations harboring specific auxotrophisms leading to slow growth and small colonies formation; SCVs also easily revert to the original wild-type phenotype depending on environmental conditions and are sometimes persisters (Tuchscherr et al., 2020). Antibiotic persistence is also distinct from resistance, characterized by an increase in the minimal inhibitory concentration (MIC) but maintenance of the bacterial capacity to multiply as long as the antibiotic concentration is kept below its current MIC. Moreover, resistance phenotypes are often genetically inherited and poorly reversible upon antibiotic withdrawal. Yet, it has been proposed that persisters may constitute an evolutionary reservoir from which resistance can emerge (Cohen et al., 2013; Windels et al., 2019). Conversely, tolerance, characterized by a reduced killing rate of bacteria (as also observed in persisters), can interact synergistically with resistance to help bacteria escaping antibiotic killing by combining these strategies (Ortiz-Padilla et al., 2020).

Persisters are also associated with specific modes of life in which bacteria show apparent unresponsiveness to antibiotics as long as they are maintained in the corresponding specific environment. Focusing on intracellular infection, the presence of persisters within the host cells may be a key determinant in the intracellular survival of small but significant inocula for many bacteria (Fisher et al., 2017). We and others have further documented that antibiotics are unable to eradicate intracellular *Staphylococcus aureus* even upon exposure of the host cells to high concentrations (typically 100-fold their MIC) of highly bactericidal agents (Krut et al., 2004; Barcia-Macay et al., 2006; Lemaire et al., 2011; Peyrusson et al., 2019), leaving apparently untouched about 0.1-1% of the original inoculum (Peyrusson et al., 2019). This contrasts with the observed extracellular activity of these drugs, which are capable of bringing CFU counts below the limit of detection in a very short time and often at concentrations that are only a few multiples of their MIC. Using the *S. aureus* laboratory strain SH1000, we recently showed that intracellular survivors collected from macrophages or monocytes exposed to high antibiotic concentrations are actually persisters (Peyrusson et al., 2020). This conclusion was supported by the demonstration of a non-dividing state for these survivors as long as they remained intracellular and under antibiotic pressure, but a rapid reversion to a growth state as soon as the antibiotic pressure was relieved; it was accompanied by an in-depth transcriptomic analysis revealing an activation of the stringent response, the cell wall stress stimulon, and the SOS and heat shock responses, together with profound changes in metabolic fluxes. However, the occurrence and quantification of persisters in clinical isolates, as well as the impact of their relative abundance on (i) evolution to resistance of the whole population and (ii) lack of eradication from phagocytes exposed to large concentrations of an active antibiotic have so far not been established.

In an attempt to address these unanswered questions, we assembled 36 non-duplicated isolates from patients hospitalized in Hanoi, Vietnam, and presenting infections that did not resolve or reactivated after 5 days of treatment with an active antibiotic (Nguyen et al., 2020), complemented with 18 randomly selected clinical isolates from the Belgian reference center for Staphylococci to give a more generalizable character to this collection. We first looked for the relative abundance of persisters in stationary cultures of these isolates, using the fluoroquinolone moxifloxacin as selecting agent based on its highly bactericidal activity against susceptible *S. aureus* in broth, and as being also one of the most active antibiotics against intracellular susceptible *S. aureus* invading and thriving in human permissive monocytes (Barcia-Macay et al., 2006; Lemaire et al., 2011; Peyrusson et al., 2020). We then compared isolates with low vs. high persisters abundance (referred henceforth as isolates with a high- and low-persister character, respectively) (i) for evolution to resistance to moxifloxacin (MIC increase) upon serial exposure to subinhibitory concentrations of this antibiotic in broth (a model previously developed and applied to the study of evolution of pneumococcal resistance to fluoroquinolones (Avrain et al., 2007), and (ii) for determination of the eradication of phagocytized bacteria from permissive monocytes exposed to moxifloxacin [using a suitable pharmacodynamic approach (Buyck et al., 2016)], together with an analysis of the capacity of survivors to divide intracellularly. All moxifloxacin-resistant isolates in the collection had a high-persister character while most but not all susceptible isolates showed a low-persister character. Most interestingly, susceptible isolates with a high-persister character evolved more rapidly to resistance in broth than those with a low-persister character. They were also lesser efficiently eradicated from phagocytes exposed to moxifloxacin (independently of their resistance pattern), with all intracellular survivors showing evidence of minimal division. Collectively, the data suggest that a high-persister character in a clinical isolate of *S. aureus* may be a contributing factor to therapeutic failures (Garzoni and Kelley, 2009; Conlon, 2014; Dubourg et al., 2017; Boudjemaa et al., 2018).

## Materials and Methods

### Main Products

Moxifloxacin HCl (potency: 90.9%) was obtained from Bayer HealthCare (Leverkusen, Germany); gentamicin sulfate (potency: 60.7%) and cation-adjusted Muller Hinton broth (CA-MHB), from Sigma-Aldrich (St. Louis, MO, United States); human serum, from Biowest SAS (Nuaillé, France); cell culture media, from Gibco/Life Technologies Corporation (Paisley, United Kingdom); Mueller Hinton broth (MHB) and tryptic soy agar (TSA), from VWR (Radnor, PA, United States); and primers, from Eurogentec (Liège, Belgium).

### Bacterial Strains and Antibiotic Susceptibility Testing

*Staphylococcus aureus* ATCC 25923 and RN 4220 are reference strains from the American Tissue Culture Collection (ATCC), Manassas, VA, United States. Thirty-six clinical isolates were collected at the Bach Mai Hospital (Hanoi, Vietnam) from patients with infections that did not resolve after 5 days of treatment by antibiotics to which the initial isolates were reported as susceptible or that reactivated after treatment discontinuation (Nguyen et al., 2020). Eighteen isolates from the collection of the Belgian National Reference Centre for Staphylococci were randomly selected and included to give a more general character to our observations. Supplementary Table 1 shows, for all these strains, their MSSA/MRSA phenotype, the MIC of moxifloxacin [determined by broth microdilution (according to the Clinical and Laboratory Standards Institute recommendations)], the moxifloxacin relative persister fraction (RPF; see next paragraph), and the corresponding assigned phenotype.

### Determination and Quantification of Persistence *in vitro*

Bacteria were grown to stationary phase after 2 sub-cultures in CA-MHB and then exposed to moxifloxacin at 100× MIC or its vehicle (sterile water; control) in CA-MHB for 5 h at 37°C under gentle rotary shaking (130 rpm). CFUs were counted after plating and overnight incubation of serially diluted aliquots. The percentage of persisters was calculated as the ratio of the CFU number in each moxifloxacin-treated sample to that in the corresponding control, and the RPF, as the ratio of the percentage of persisters for each clinical isolate to that observed for ATCC 25923 (De Groote et al., 2009).

### *In vitro* Selection of Resistance

Bacteria from an overnight broth culture were inoculated in 12-well plates (0.5 × 10^6^ CFU/mL) in CA-MHB and exposed to moxifloxacin at 0.5× MIC, with daily measurement of the MIC and readjustment of the moxifloxacin concentration to half of this value until the MIC reached a value of 2 mg/L (Avrain et al., 2007). This experimental set-up referred to as “serial passage” is widely used to follow evolution toward resistance (Martinez et al., 2007) especially when it is related to mutations in target genes or gene regulators. It does not mimic what could occur in clinical practice when patients are exposed to optimal dosing regimens, but rather in case of antibiotic underdosing known as a main driver of evolution toward resistance (Heffernan et al., 2018).

### Determination of Gene Expression Levels by RT-PCR

Stationary-phase cultures were incubated with moxifloxacin or its vehicle as for the persister assay, and harvested by centrifugation either immediately or after incubation with moxifloxacin at 100× MIC. Bacteria were lysed with lysozyme and lysostaphin, after which RNA was isolated (InviTrap^®^ Spin Universal RNA Mini Kit; Stractec, Berlin, Germany) and purified (TURBO DNA-free^TM^ Kit; Invitrogen, Carlsbad, CA, United States). The RNA quality and quantity were determined by spectrophotometry (NanoDrop^TM^, Thermofisher, Waltham, MA, United States). Purified RNA was converted to cDNA (Transcription first strand cDNA synthesis kit; Roche Applied Science, Penzberg, Germany). Quantitative RT-PCR was performed with a iCycler^TM^ iQ instrument (Bio-Rad, Hercules, CA, United States) using 5 μL of cDNA, 12.5 μL of SYBR Green Master Mix (Bio-Rad), 2 μL of 5 mM of each primer and 3.5 μL of sterile RNase-free water (Ambion, St. Austin, TX, United States). PCR program steps were (i) 95°C 3 min (denaturation) (ii) 40 cycles (95°C 15 s–61°C 60 s) (amplification). Primers are described in Supplementary Table 2. A melting curve was run to check for the presence of a unique PCR reaction product. Relative expression levels were calculated by the ΔΔCt method, with *gmk* as housekeeping gene and *S. aureus* ATCC 25923 as a reference.

### Screening for Mutations in the Genes Encoding Fluoroquinolone Targets

Template DNA was purified with GeneJET Genomic DNA Purification Kit (Thermofisher). PCR amplification of the QRDRs of *gyrA*, *gyrB*, *parC*, and *parE* was performed using 1 μL of DNAg, 5 μL of 5× Phusion buffer, 0.8 μL of 10 mM dNTPs, 1 μL of 10 mM of each primer (see Supplementary Table 2), 0.25 μL of Phusion polymerase (Thermofisher) and 15.95 μL sterile nuclease-free water (Ambion). The PCR program steps were (i) 98°C 3 min (denaturation), (ii) 30 cycles [98°C 10 s–60°C 30 s (56°C 30s with *parE*), 72°C 60 s] (amplification), and (iii) 72°C 10 min (full amplicons extension). Sanger sequencing was performed in forward and reverse directions at Genewiz (Leipzig, Germany) using the same primers. QRDR DNA sequences were compared with those of *S. aureus* RN4220.

### Intracellular Activities of Antibiotics

THP-1 human monocytes (clone ATCC TIB-202; American Type Culture Collection) were infected with *S. aureus* as previously described, and non-phagocytized bacteria eliminated by short exposure (45 min) to gentamicin at 50 to 100× its MIC (thus limiting the study to isolates for which the MIC of gentamicin was ≤ 8mg/L (i.e., a maximum of 4-fold the current EUCAST cut-off of 2 mg/L observed for wild type strains) (Barcia-Macay et al., 2006). Results are expressed as the change in the number of CFU per mg of cell protein after 24 h incubation of infected cells with moxifloxacin. We checked in preliminary experiments that moxifloxacin, including at high concentrations, did not affect the protein content of the samples neither the number of cells after 24 h of incubation. We also checked that proteins from dead cells do not interfere in the protein assay (signal barely distinguishable from that of the blank for cells killing by incubation with DMSO or Triton X-100). The activity of moxifloxacin against intracellular bacteria was measured using full concentration-response experiments (change in intracellular CFU from the post-phagocytosis inoculum plotted against the extracellular antibiotic concentration [both in log_10_ units] typically ranging from 10^–2^ to 10^+2^ fold the MIC). A Hill-Langmuir equation [with slope factor set to 1 as discussed previously (Lemaire et al., 2009; Peyrusson et al., 2019)] was fitted to the data to calculate two key pharmacodynamic parameters [apparent static concentration (C_s_) and maximal relative efficacy (E_max_); see Buyck et al. (2016) and in the caption of Figure 4].

### Transformation of *Staphylococcus aureus*

*gfp*, encoding the Green fluorescent protein (GFP), cloned with the *xyl/tetO* inducible promoter carried on a shuttle pALC 2084 *Escherichia coli* – *S. aureus* plasmid was used as a reporter (Bateman et al., 2001) transformed into *S. aureus* by electroporation following exactly a previously described protocol (Rhoads Kraemer and Iandolo, 1990). Colonies were checked for GFP expression (fluorescence microscopy) after incubation with 0.125 mg/L tetracycline (inducer), and for unimpaired susceptibility to moxifloxacin, gentamicin, and chloramphenicol.

### Flow Cytometry

Transformed bacteria were cultured overnight in CA-MHB supplemented with 0.125 mg/L tetracycline (GFP inducer) and 10 mg/L chloramphenicol. They were used for intracellular infection following our general protocol, except that the phagocytosis time was reduced to 30 min (to limit bacterial division during this step), and the ratio of bacteria to THP-1 cells doubled (to ease the fluorometric quantitation of the intracellular inoculum). Tetracycline was removed from the medium at the end of the phagocytosis step. CFUs counting and FACS analysis were performed in parallel on the same samples as previously described (Peyrusson et al., 2020). In brief, bacteria isolated from THP-1 cells were resuspended in filtered PBS, stained with 10 mg/L propidium iodide, and analyzed using a FACSVerse cytometer (BD Biosciences, Franklin Lakes, NJ, United States) for GFP signal intensities (FITC channel, medium flow rate). Forward-scatter width (FCS-W) vs. forward-scatter area (FSC-A), and side-scatter width (SSC-W) vs. side-scatter area (SSC-A) were used to gate out damaged (propidium iodide-positive) or multiplet bacteria (Peyrusson et al., 2020). Data were analyzed with FlowJo 10.5.2 software (TreeStar Inc., Ashland, OR, United States).

## Results

### Determination of the Moxifloxacin-Persister Character of Clinical Isolates

**FIGURE 1:**
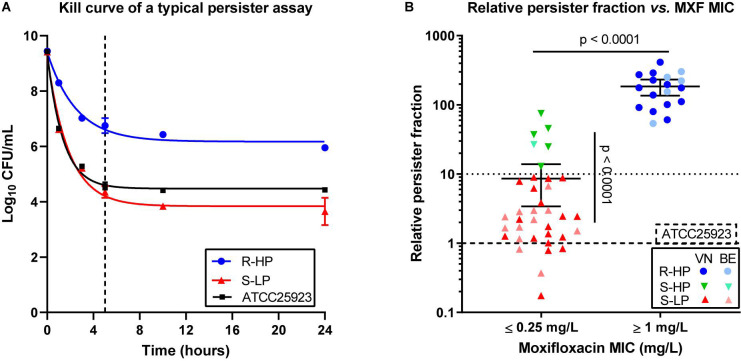
Persister character of clinical isolates. **(A)** Illustration of the persister assay in stationary phase cultures for ATCC 25923 (reference strain; black symbols and curve), one typical susceptible isolate with low-persister character (S-LP; 69687; red symbols and curve), and one typical resistant isolate with high-persister character (R-HP; 13890; blue symbols and curve). The graph shows the reduction in CFUs over time of incubation with moxifloxacin at 100 × MIC. The data were used to fit one phase exponential decay with plateau functions (*R*^2^ = 0.99, 0.98, and 0.98). The dotted vertical line shows the time point (5 h) selected for performing the persister assay in the whole collection. **(B)** Relative persister fraction of each isolate stratified according to its susceptibility to moxifloxacin using EUCAST interpretive criteria (susceptible: MIC ≤ 0.25 mg/L). The graph shows the relative persister fraction [% persisters for the isolate/% persisters for ATCC 25923 (tick dotted line)]. A low-persister phenotype was attributed to isolates with a persister fraction ≤10 (thin dotted line) and a high-persister phenotype, to isolates with a persister fraction >10. On this basis, 3 subgroups were defined, namely susceptible isolates with low-persister fraction (S-LP, in red), susceptible isolates with high-persister fraction (S-HP, in green), and resistant isolates with high-persister fraction (R-HP, in blue), with dark and light colors corresponding to isolates from Vietnam (VN) or Belgium (BE), respectively. Individual data are shown together with the mean value and the 95% confidence interval. Persister tests were performed in 3 independent experiments each run in duplicate, the mean value for each isolate corresponding to one symbol. Statistical analysis: Mann-Withney test comparing (a) all moxifloxacin susceptible (MIC ≤ 0.25 mg/L) vs. all moxifloxacin-resistant (MIC ≥ 1 mg/L) isolates (no isolate with a MIC of 0.5 mg/L in this collection) or (b) S-LP vs. S-HP isolates: *p* < 0.0001. Kruskal–Wallis test comparing these three subgroups shows that the persister fraction is significantly different between S-LP and S-HP, but not between S-HP and R-HP (not shown on the graph).

Figure 1A shows the killing of ATCC 25923 and 2 selected Vietnamese clinical isolates in stationary phase cultures over time of exposure to moxifloxacin at 100× MIC. A plateau of killing [typical of persisters (Balaban et al., 2019)] was reached after about 5 h. Figure 1B shows the RPF for all Vietnamese and Belgian isolates, categorized according to their susceptibility to moxifloxacin (individual values in Supplementary Table 1). Among moxifloxacin-susceptible isolates, we could categorize the isolates as having a low (≤10; *n* = 30/36) or a high (>10; *n* = 6/36) RPF. All moxifloxacin-resistant isolates (*n* = 18) showed a high RPF. We therefore considered three phenotypic subclasses for this work, henceforth referred to as susceptible isolates with low relative persister fractions (S-LP), or with high relative persister fractions (S-HP), and resistant isolates with high relative persister fractions (R-HP), respectively^1^.

### Expression of Genes Involved in SOS Response and Encoding Fluoroquinolone Targets

**FIGURE 2:**
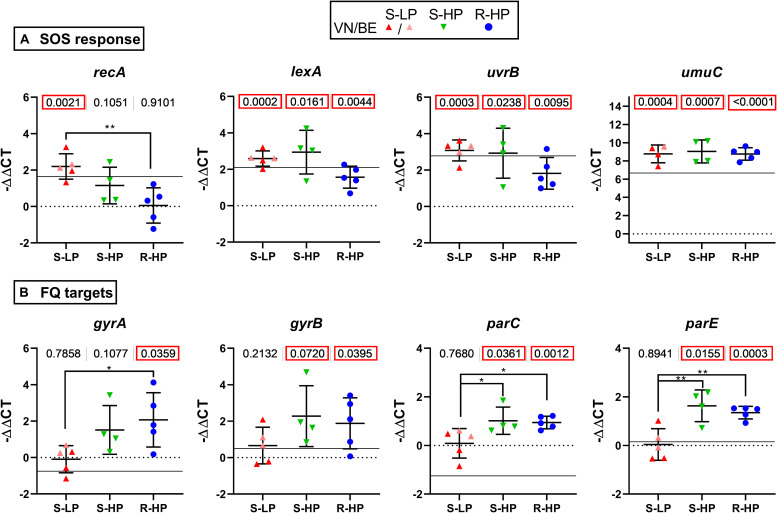
Gene expression and influence of moxifloxacin. Expression of genes encoding proteins involved in SOS response (**A**) or fluoroquinolone targets (**B**) in bacteria incubated for 5 h with moxifloxacin at 100 × MIC in the conditions used for performing the persister assay. Data are for selected isolates with susceptible low-persister [S-LP, red (30337, 69474, and 69687)], susceptible high-persister [S-HP, green (69505, 69783, and 69867)], or resistant high-persister [R-HP, blue (30462, 69781, 30566, 13890, and 35994)] phenotypes. Each dot represents one isolate (mean value of triplicates), with the global mean and SD. Data are expressed as −ΔΔCT, with the value of ATCC 25923 at time 0 h used as a reference (horizontal dotted line). The thin horizontal line shows the expression level measured for ATCC 25923 at 5 h. Statistical analysis: the figures at the top of the graphs shows the *p* value of a *t*-test comparing the group of isolates with ATCC 25923 at time 0 h. *p* values lower than 0.05 are squared in red. Stars highlight differences among subgroups by ANOVA analysis (Tukey *post hoc* test): *p < 0.05; **p < 0.01.

Exposure of bacteria to fluoroquinolones triggers a SOS response, which, in turn, increases mutation frequency (Qin et al., 2015) and development of resistance, but also induces persistence (Dorr et al., 2009). Using representative isolates of the three phenotypic groups defined above and exposed to moxifloxacin as for the persister assay, we measured the expression of the SOS response regulon repressor *lexA*, the derepressor *recA*, and two genes of this regulon [*uvrB*, encoding the excision nuclease subunit B recognizing DNA lesions and involved in repair processes initiated by fluoroquinolone-induced damage; *umuC*, encoding an error-prone polymerase introducing mutations in DNA during replication (Qin et al., 2015; Maslowska et al., 2019)]. The expression of these genes was induced in all isolates, except for *recA* (overexpressed in S-LP, to a lesser extent in S-HP, but not in R-HP; Figure 2A). Because genes encoding fluoroquinolone targets are potentially regulated by the SOS response (Cirz et al., 2007), we measured their expression in the same conditions (Figure 2B). We observed an overexpression in S-HP and R-HP but not in S-LP.

### Selection of Resistance to Moxifloxacin in Susceptible Isolates With Low- and High-Relative Persister Fractions

**FIGURE 3:**
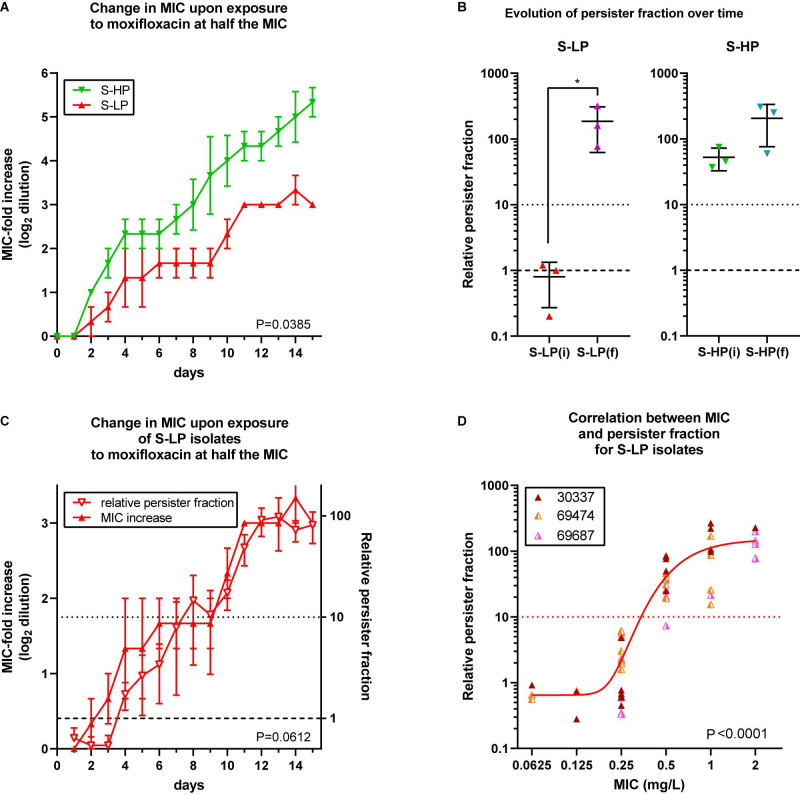
Selection of resistance in planktonic cultures from high-persisters and low-persister isolates. **(A)** Increase in MIC (expressed in log_2_ fold dilution) over time of incubation with moxifloxacin at 0.5 × MIC over 15 subcultures, with readjustment of the moxifloxacin concentration at every passage according to its measured MIC. Data are the means ± SEM of 2 independent experiments with three selected isolates showing a susceptible low-persister phenotype [S-LP, red (30337, 69474, and 69687)] or high-persister phenotype [S-HP, green (69505, 69783, and 69867)] phenotype at day 0. **(B)** Relative persister fraction in initial S-LP [S-LP(i)] and S-HP [S-HP(i)] isolates and in resistant mutants thereof collected when having reached an MIC of 2 mg/L [S-LP(f) and S-HP(f)]. **(C)** Increase of moxifloxacin MIC (left ordinate) and in relative persister fraction (right ordinate) for S-LP isolates over 15 days. **(D)** Relationship between the relative persister fraction and the moxifloxacin MICs for S-LP isolates subjected to the resistance selection process **(A)** and collected at various times to obtain isolates for which the moxifloxacin MICs spanned between 0.0625 and 2 mg/L [the curve is an asymmetrical Hill function (5 parameters sigmoidal function) fitted to the data; *R*^2^ = 0.61]. Statistical analysis: **(A,C)**: 2-way ANOVA; the *P* value shown on the graph is the one calculated for the comparison of the two phenotypic groups. **(B)**: Paired *t*- test. **(D)**: Pearson correlation coefficient = 0.7336; corresponding *P* value shown on the graph.

Since all moxifloxacin-resistant isolates showed high relative persister fractions, we tested whether S-HP isolates would be more prone to develop resistance than their S-LP counterparts. We used a previously developed model (Avrain et al., 2007) in which bacteria are exposed to subinhibitory concentrations of a fluoroquinolone in a closed system (excluding therefore the acquisition of foreign genetic material), to specifically assess the intrinsic ability of isolates for spontaneous evolution to develop resistance while avoiding selection of pre-existing subpopulations with higher MIC. Thus, 3 S-LP and 3 S-HP isolates were exposed to 0.5× MIC of moxifloxacin and the change in MIC measured (and moxifloxacin concentration readjusted) each day. Figure 3A (with data limited to the first 15 days of the experiment) shows that resistance was acquired faster for S-HP than for S-LP [mean time for onefold MIC increase, using data from day 2 through 15: 2.44 ± 0.37 vs. 4.34 ± 0.77 days (*n* = 2 × 14; *p* < 0.0001; two tailed unpaired *t*-test with Welsh’s correction for unequal SD’s)]. At the end of the selection process, mutants (with an MIC of 2 mg/L) had a RPF of approx. 100 [significantly increased for S-LP only, to levels similar to those observed in R-HP (Figure 3B)]. The RPF and moxifloxacin MIC increased in parallel in S-LP during this process (Figure 3C), with all strains with a MIC ≥ 0.5 mg/L showing a RPF > 10 (Figure 3D). We also evaluated in these mutants the expression of genes involved in SOS response and encoding fluoroquinolone targets. They were all overexpressed to levels similar or even higher than in their parental isolates (Supplementary Figure 1).

### Mechanisms of Resistance to Moxifloxacin

Supplementary Table 3 shows the mutations observed in the QRDR of *gyrA*, *gyrB*, *parC*, and *parE* of selected isolates from the three phenotypic groups and of resistant mutants selected *in vitro* from S-LP and S-HP. In most susceptible isolates, we noticed a Glu422Asp substitution in ParE, known as not causing resistance (Schmitz et al., 1998). Isolate 69687, which showed a more elevated moxifloxacin MIC, harbored a His103Tyr mutation in ParC, previously described as frequently associated with other mutations in resistant strains (Morrow et al., 2011). All moxifloxacin-resistant clinical isolates presented a Ser84Leu mutation in GyrA and a Ser80Phe mutation in ParC, which together are known as conferring resistance (Roychoudhury et al., 2001; Kwak et al., 2013) and are described in isolates for which moxifloxacin MICs are in the 1–4 mg/L range (Schmitz et al., 1998; Nakaminami et al., 2014). For moxifloxacin-resistant mutants obtained from S-LP isolates, mutations in GyrA (Ser84Leu), GyrB (Leu433Ile or Ile454Val), or ParC (Ser80Phe or Ser80Ala) were detected. To our knowledge, these mutations in GyrB or the substitution of Ser80Ala in ParC have not been previously described. Thus, each of the final mutants showed two mutations in fluoroquinolone targets, which is what is generally required for moxifloxacin to reach MICs ≥ 1 mg/L (Noguchi et al., 2005; Bhagwat et al., 2006). For moxifloxacin-resistant mutants obtained from S-HP, only one mutation in GyrA (Ser80Leu) was detected, which was previously found in rare resistant isolates (Kwak et al., 2013).

Genes encoding efflux transporters *norA*, *norB*, and *norC* were slightly overexpressed in H-RP, and *norB*, in *in vitro* selected mutants, after incubation with moxifloxacin at 100× MIC, when compared to ATCC 25923 (Supplementary Figure 2).

### Quantitative Measurement of Intracellular Activity of Moxifloxacin

**FIGURE 4:**
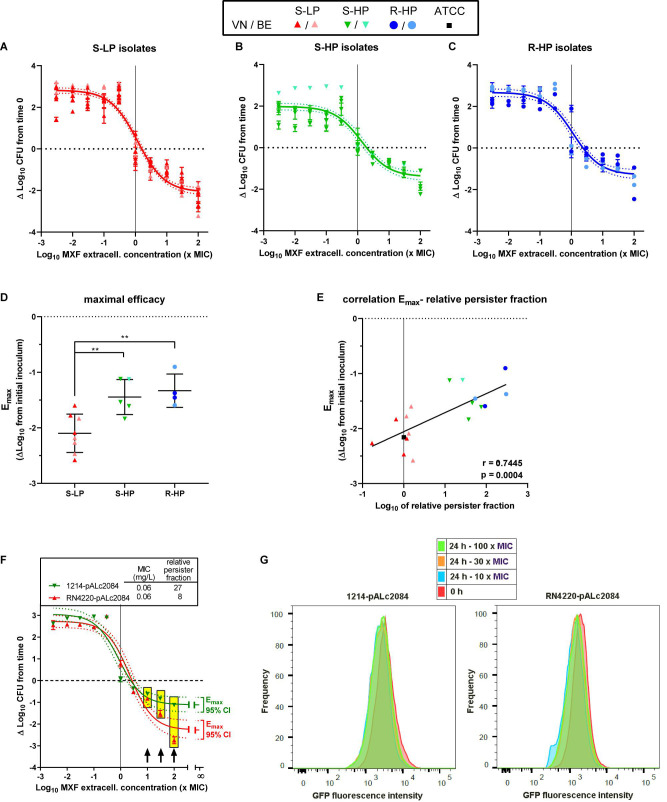
Intracellular activity of moxifloxacin. Top **(A–C)** Concentration-response curves of the activity of moxifloxacin against intraphagocytic *S. aureus* with different resistance/persistence phenotypes [**(A)**: isolates 30337, 69474, 69687, 34467, 2017S228, 2016S354, 2014S118, and 1078 with susceptible low-persister character (S-HP); **(B)**: isolates 69505, 69783, 69867, 36606, and 1214 with susceptible high-persister character (S-HP); **(C)**: isolates 20975, 13890, 2015S418, and 2017S017 with resistant high-persister (R-HP) character]. Infected THP-1 monocytes were incubated for 24 h with the antibiotic at concentrations (expressed in × MIC of each individual isolate) covering a large span to allow for a full concentration-response analysis (see Buyck et al., 2016). The horizontal dotted line shows the initial post-phagocytosis inoculum. Data are expressed as changes from this value after 24 h of incubation. The dots on each graph correspond to experimental values for individual isolates (with their respective SD) sharing the same resistance-persistence phenotype and the curve is a single Hill-Langmuir function (with slope factor set to 1) fitted to the data of all isolates in each group (with the dotted lines corresponding to its 95% confidence interval; see Supplementary Table 4 (Supplementary Material) for individual Hill-Langmuir function parameters of interest [E_*max*_ (maximal relative efficacy) and IC_50_]. The vertical line points to the MIC. Middle **(D)** Maximal relative efficacy (E_*max*_) of moxifloxacin (reduction in intracellular CFUs as extrapolated for an infinitively large antibiotic concentration based on the Hill equation parameters determined for each individual strain). The dots are the values of each individual isolate; the horizontal lines, are the mean and SD of each phenotypic group. Statistical analysis: one-way ANOVA (*p* = 0.0025) with Tukey *post hoc* test comparing the three phenotypic subgroups: **p < 0.01; there was no significant difference between the S-HP and R-HP groups. **(E)** Correlation between the intracellular E_*max*_ (determined as shown here) and the relative persister fraction [as determined in stationary phase culture (see Supplementary Table 1), using log scale for both. The vertical dotted shows the relative persister fraction of the reference ATCC 25923. *r* and *p*: Pearson coefficient and *p* value of the correlation. Bottom **(F)** Concentration-response curves of the activity of moxifloxacin against intraphagocytic RN4220 (S-LP) and 1214 (S-HP) transformed by the pALc2084 plasmid to express GFP under induction by tetracycline (maintained till the end of the phagocytosis period), as measured after 24 h of incubation of the infected cells in the presence of moxifloxacin over a wide range of concentrations (expressed in × MIC of each individual isolate) but in the absence of tetracycline. The Hill-Langmuir function (with slope factor set to 1) fitted to the data of each isolate is shown, together with the E_*max*_ of each curve with its 95% confidence interval at an extrapolated infinitively large concentration. The horizontal dotted line shows the initial post-phagocytosis inoculum; data are expressed as changes from this value after 24 h of incubation. The vertical line points to the MIC. The table above the graph shows the MIC and relative persister fraction of these strains. Yellow rectangles highlight samples that were examined in FACS, with the corresponding concentrations shown by black arrows. **(G)** Flow cytometric profiles of bacteria recovered from monocytes at the end of the phagocytosis period (0 h) or after 24 h of incubation with moxifloxacin at the indicated concentrations. The graphs show flow cytometric profiles of the frequency of events as a function of GFP fluorescence intensity.

In a next step, we evaluated the intracellular activity of moxifloxacin against isolates representative of the S-LP, S-HP, or R-HP phenotypes [selected based on a gentamicin MIC ≤ 8 mg/L; see methods and Barcia-Macay et al. (2006)]. The post-phagocytosis inoculum was similar among the three phenotypic groups [6.4 ± 0.1, 6.5 ± 0.2, and 6.7 ± 0.4 log_10_ CFU/mg protein for isolates with S-LP, S-HP, and R-HP character, respectively (not statistically different by one-way ANOVA)]. Figures 4A–C shows the moxifloxacin patterns of activity in concentration-response experiments after 24 h of incubation with equipotent antibiotic concentrations (same multiples of the MIC). A Hill-Langmuir function could be fitted to all data, with a single equation for all isolates within each phenotypic group. The static concentrations (C_s_) were close to the corresponding MICs, denoting similar relative potencies. In contrast, the moxifloxacin maximal relative activity [E_max_, which the model fitted to the data determines by extrapolation of the sigmoid function for an infinitely large antibiotic concentration (describing, therefore, the maximal amplitude of the bacterial response to the antibiotic)], was significantly lower (less negative) for S-HP or R-HP than for S-LP [−1.45 ± 0.31 and −1.33 ± 0.30 vs. −2.10 ± 0.34 log_10_ CFU (Figure 4D); these values, which correspond to a bottom plateau in the function, are actually close to those measured at the highest extracellular moxifloxacin concentration tested]. An inverse correlation was also observed between the E_max_ value and the RPF of each isolate (Figure 4E). Thus, these data show that cell infection by isolates with high relative persister fractions results in larger intracellular residual inocula that remained unaffected by moxifloxacin compared to what is observed for an isolate with a low relative persister fraction. In addition, we found that the growth rate of bacteria collected from intracellular experiments and reinoculated in fresh broth was similar to that of bacteria from fresh culture or collected after the persister assay (Supplementary Figure 3), suggesting a reversion to a normal phenotype once the antibiotic pressure has been removed.

### FACS Analysis of Intracellular Persisters

The S-LP (RN4220) and S-HP (1214) isolates expressing GFP under the control of a tetracycline-inducible promoter behaved intracellularly as clinical isolates, with C_*s*_ at values close to their MIC, and a lower (less negative) E_max_ of moxifloxacin when infected with S-HP than L-HP (−1.1 vs. −2.3 log_10_ CFU; Figure 4F). FACS analysis shows that propidium-negative (viable) bacteria collected from both strains after 24 h exposure to moxifloxacin at high concentrations remained highly fluorescent, indicating that they did not divide intracellularly (Figure 4G).

## Discussion

This study examines, in clinical *S. aureus* isolates exposed to the fluoroquinolone moxifloxacin, the correlation between (i) their high- vs. low-persister character (as determined by the relative abundance of persisters isolated from stationary cultures exposed to moxifloxacin), (ii) evolution to resistance (by spontaneous mutations upon exposure to subtherapeutic concentrations of the same antibiotic), and (iii) intracellular survival in permissive host cells in spite of exposure of these infected cells to high moxifloxacin concentrations. Isolates were from two geographically distinct origin (Vietnam and Belgium) to gain in clinically pertinent information and value. Conversely, we restricted this first analysis to a single antibiotic, taken as pharmacological tool, to minimize drug-related differences when addressing different biological and potentially disease-related properties of persisters. Moxifloxacin was selected as a highly bactericidal antibiotic, fulfilling therefore the condition set for evidencing the occurrence of persisters (Balaban et al., 2019). In our set-up, it also offered the advantage of being clinically approved for the treatment of staphylococcal infections, while showing a balanced proportion of susceptible and resistant clinical isolates [a condition that becomes increasingly difficult to meet with other antibiotics when dealing with hospitalized patients, as exemplified here by our previous analysis of the resistance patterns of the Vietnamese collection (Nguyen et al., 2020)]. Three key observations are presented, which collectively may help in understanding how persistence to antibiotics may eventually account for clinical failures in spite of well-conducted antibiotic treatments.

First, the study shows a correlation between the ability of strains to give rise to persisters (quantitated here by their relative persister fraction) and their level of resistance to moxifloxacin. We indeed found that moxifloxacin-resistant isolates have all a high-persister character, while this character is more variable among susceptible isolates. This observation, as far as we know, has never been made using a large collection of clinical isolates. Antibiotic persistence and resistance are considered as distinct phenotypes leading independently to therapeutic failure (Fisher et al., 2017; Balaban et al., 2019). We show here, for moxifloxacin, that these two phenotypes are actually closely related and that resistance develops more rapidly in isolates with a high- rather than a low- relative persister fraction when exposed to subinhibitory concentrations of this antibiotic. Studies with laboratory strains of *E. coli* have demonstrated that persistence promotes evolution to resistance by increasing survival and mutation rates (Barrett et al., 2019; Windels et al., 2019). We extend this conclusion to *S. aureus* and clinical isolates. In this context, we add the observation that the expression of *recA*, the derepressor of the SOS response, is not induced in resistant isolates, but well in *in vitro* selected mutants, suggesting that the SOS response is involved in resistance acquisition but is no longer necessary once resistance has been acquired (Torres-Barcelo et al., 2015; Machuca et al., 2017). As expected, the main mechanism of resistance selected consisted in mutations in fluoroquinolones targets, with ParC and GyrA being most frequently affected, as in most other studies (Schmitz et al., 1998; Roychoudhury et al., 2001; Kwak et al., 2013; Nakaminami et al., 2014).

In this context, a second key observation is that moxifloxacin induces the expression of genes encoding fluoroquinolone targets, especially in isolates with a high-persister character. This occurred independently of a decrease in susceptibility as well as its actual level, suggesting that this overexpression is associated with acquisition of a persister phenotype rather than of resistance. This is coherent with our recent observation that *gyrA* and *gyrB* are overexpressed in intracellular *S. aureus* persisters as part of a global response to the stress imposed by antibiotics, including by other classes than fluoroquinolones (Peyrusson et al., 2020).

A third major finding of this work is that isolates yielding a large proposition of persisters in stationary phase culture (broth) and phagocytized by THP-1 monocytes gave rise to larger residual inocula that remained unaffected by moxifloxacin intracellularly (less negative E_max_, which refers to the decrease in CFU’s for an infinitely large extracellular concentrations, therefore denoting the drug maximal activity) compared to isolates categorized as showing a low-persister character in the same broth assay. We recently demonstrated for a laboratory strain of *S. aureus* that bacteria surviving to antibiotics intracellularly are persisters, showing a non-dividing phenotype readily reversible as soon as the antibiotic pressure is relieved (Peyrusson et al., 2020). We show here that this conclusion can be applied to clinical isolates as well, which opens a number of medically important avenues to explore. We also bring a rationale as why the intracellular maximal relative efficacy of a given antibiotic may vary among clinical isolates (Lemaire et al., 2011), if considering their potential differences in relative persister fraction. Thus, a high-persister character could stand as an important cause of ineffective elimination of intracellular *S. aureus*.

We also need to comment on the possible relationship between the presence of persisters among clinical isolates of *S. aureus* and persistence and/or recurrence of staphylococcal infections. Persisters are actually observed for all isolates from both the Vietnamese and Belgian collection (though in variable proportions), representing 0.001–1% of the population in stationary cultures if defined as cultivable organisms that remain unaffected by moxifloxacin at 100× its MIC. Conversely the residual intracellular inocula observed in cells exposed to similarly large concentrations of moxifloxacin represent from 1 to 10% of the post-phagocytosis inoculum, with most if not all being non-dividing, and therefore fulfilling in both respects the definition of persisters. This suggests a drastic enrichment in persisters when isolates are exposed to antibiotics in the intracellular milieu. If fractional, this effect of the antibiotics is anticipated to give rise to larger intracellular populations of persisters if infection is made with isolates that are producing more persisters in stationary phase cultures (and were categorized as having a high persister character). This is exactly what we found when comparing the sizes of the surviving intracellular pools (denoted by the differences in E_max_ values), since these survivors were more numerous if the experiment was performed with isolates with high relative persister fraction rather than with those with low relative persister fraction, even though the initial post-phagocytosis inocula were not different. This fully supports and strengthen the concept that one of the factors leading to intracellular survival in spite of exposure of the host cells to high antibiotic concentrations is the persister character of the isolate. Persisters are often considered as pre-existing to antibiotic exposure and be related to an ill-adapted immune host response or to the presence of growth-arrested bacteria (Zhang, 2014; Fisher et al., 2017). But we see here that the proportion of persisters is increased when the antibiotic exposure has taken place intracellularly. This, therefore, also suggests that the mechanisms responsible for the emergence of the persister phenotype involves more than pre-existing populations. It also reinforces our suggestion that persisters surviving antibiotic treatment intracellularly may contribute to relapses by recovering their capacity to divide and escape from the host cells once antibiotic therapy is discontinued (Fisher et al., 2017), since we see here that intracellular niches may result in considerable enrichment of the initial inocula with what are probably bacteria untreatable by currently available antibiotics. Resistance, however, remains important since moxifloxacin, at an extracellular concentration corresponding to its human C_max_ (4 mg/L), had only a bacteriostatic effect on intracellular R-HP *S. aureus* isolates.

This study has limitations. First, we examined only a single antibiotic (moxifloxacin), which, however, was not only by design, as explained earlier, but also for feasibility reasons, having made the choice to work not with a limited number of strains but with a large collection of isolates to obtain conclusions that could generalized to real-life samples and be potentially applicable to the clinic. Other fluoroquinolones could have been added to the study, but active and widely used anti-staphylococcal fluoroquinolones are few amongst the approved members of this class of antibiotics. Conversely, other bactericidal antibiotics from different pharmacological and/or chemical classes will need to be included in future investigations to reach truly generalizable conclusions. Of interest in this context, we recently observed that intracellular persisters can not only be selected by exposure to antibiotics belonging to different classes but also show unresponsiveness to multiple antibiotic classes, which can be explained by an activation the global stress responses (Peyrusson et al., 2020). A second limitation stems from the lack of comprehensive molecular studies of the mechanisms underlying the differences in persister fractions among clinical isolates, which would require in-depth transcriptomic and/or genomic analyses, requiring, for critical cases, further documentation at the protein and function levels. Both limitations call for additional studies, but represent major undertakings by them-selves.

At this stage, however, and in the limits of our current investigations, our work already opens the perspective that determining antibiotic persistence may help defining the risk of developing resistance and of maintaining a pool of dormant bacteria surviving intracellularly that may reactivate the infection at the end of the treatment. Screening for the persister character of clinical isolates, and establishing the predictive value and specificity of the results of such assay, could become an important contribution to the improvement of antibiotic treatments in the future. This would require, however, the development of routine-usable methodologies, a first example of which could be the recently published TDtest, which used a modified disk-diffusion assay in order to detect antibiotic tolerance (Gefen et al., 2017).

## Data Availability Statement

The raw data supporting the conclusions of this article will be made available by the authors, without undue reservation.

## Ethics Statement

The study did not involve directly patients, but clinical isolates that were collected prospectively in Vietnam with the approval of the ethical committee from the Hanoi University of Pharmacy (1102/QD–DHN). The Belgian isolates were randomly selected in the collection of the Belgian reference center for Staphylococci, which does not need ethical approval.

## Author Contributions

TKN performed the experiments and analyzed the data. FP performed FACS analyses. MD and NHP provided clinical isolates and characterized them. HAN and PMT provided advice and PMT reanalyzed the data in details. FVB supervised the study and wrote the Manuscript. All the authors made useful comments during the writing of the Manuscript and approved the submitted version.

## Conflict of Interest

The authors declare that the research was conducted in the absence of any commercial or financial relationships that could be construed as a potential conflict of interest.

## Footnotes

1 To avoid any ambiguity, S or R in the abbreviation of susceptible or resistant isolates refer to the MIC measured in broth and consider therefore a property attributed to (or assumed to be shared by) each individual organism in the entire population (thus, disregarding potential differences in MIC among individual organisms that could be revealed, for instance, by diffusion or population analysis techniques). Conversely, the “HP” or “LP” abbreviations used to denote isolates with a high- or a low-persister character refer to a low or high percentage of bacteria isolated from the initial population relative to the value observed with the laboratory strain ATCC 25923. It, therefore, does not mean that the persister character concerns the whole population, since actually, most of the individual bacteria are not persisters. It rather refers to a small subpopulation and describes its relative abundance in the whole population (selected during the assay) in the conditions of the assay.
