# Supplementary material for: The Persister Character of Clinical Isolates of Staphylococcus aureus Contributes to Faster Evolution to Resistance and Higher Survival in THP-1 Monocytes: A Study With Moxifloxacin
Source: Front Microbiol. 2020 Nov 23;11:587364. doi: 10.3389/fmicb.2020.587364 (PMC7719683; doi:10.3389/fmicb.2020.587364)
Supplement: Supplementary file 1 [file Data_Sheet_1.docx]

Supplementary Material

**Table S1: characteristics of the strains used in this study**

*S. aureus* ATCC 25923 and RN 4220 are reference strains from the American Tissue Culture Collection (ATCC), Manassas, VA. Thirty-six clinical isolates were collected at the Bach Mai Hospital (Hanoi, Vietnam) from patients with microbiological and clinical evidence of persisting and/or recurrent infection (defined as (i) an infection that did not resolve after 5 days of treatment by antibiotics to which the initial isolates were reported as susceptible or (ii) an infection that reactivated a few days after the administration of an antibiotic to which the initial isolate was susceptible but had been discontinued based on clinical evidence of improvement of the patient’s conditions)(Nguyen et al., 2020). Their Agr group, typing (based on *spa* [Staphylococcus protein A] type and Pulsed Field Gel Electrophoresis), and susceptibility to 26 anti-staphylococcal antibiotics (covering the main pharmacological classes) have been reported previously (Nguyen et al., 2020). Eighteen randomly-selected isolates from the collection of the Belgian National Reference Center for Staphylococci were included as complement.

| **Origin** | **Strain identification** | **MSSA/MRSA^a^** | **Moxifloxacin MIC (mg/L)^b^** | **Moxifloxacin relative persister fraction (RPF)^c^** | **Assigned phenotypic group** |
| --- | --- | --- | --- | --- | --- |
| References | ATCC 25923 | MSSA | 0.032 | 1.0 |  |
|  | RN 4220 | MSSA | 0.064 | 8.0 | Susceptible with  low– relative persister fraction  (S-LP) |
| Bach Mai hospital, Hanoi, Vietnam | 69474 | MRSA | 0.064 | 0.2 | Susceptible with  low– relative persister fraction  (S-LP) |
|  | 30549 | MSSA | 0.032 | 0.8 |  |
|  | 3446 | MSSA | 0.064 | 0.8 |  |
|  | 69687 | MRSA | 0.25 | 1.0 |  |
|  | 30337 | MRSA | 0.064 | 1.2 |  |
|  | 69493 | MRSA | 0.064 | 1.2 |  |
|  | 22714 | MRSA | 0.064 | 1.3 |  |
|  | 69613 | MSSA | 0.064 | 1.4 |  |
|  | 69486 | MRSA | 0.064 | 1.7 |  |
|  | 17883 | MSSA | 0.032 | 2.2 |  |
|  | 30461 | MSSA | 0.064 | 2.4 |  |
|  | 19255 | MSSA | 0.064 | 2.5 |  |
|  | 69700 | MRSA | 0.064 | 3.9 |  |
|  | 34613 | MRSA | 0.032 | 6.2 |  |
|  | 69544 | MRSA | 0.032 | 7.8 |  |
|  | 34724 | MRSA | 0.064 | 8.6 |  |
|  | 36271 | MRSA | 0.032 | 9.0 |  |
|  | 36606 | MSSA | 0.032 | 12.9 | Susceptible with  high– relative persister fraction  (H-LP) |
|  | 14305 | MSSA | 0.032 | 24.8 |  |
|  | 69783 | MSSA | 0.032 | 37.2 |  |
|  | 69505 | MRSA | 0.032 | 45.5 |  |
|  | 69867 | MRSA | 0.064 | 74.8 |  |
|  | 30566 | MSSA | 2 | 60.7 | Resistant with  high– relative persister fraction (R-HP) |
|  | 30342 | BORSA or MODSA | 2 | 79.8 |  |
|  | 69519 | MRSA | 1 | 91.6 |  |
|  | 20975 | MSSA | 2 | 92.7 |  |
|  | 30609 | MRSA | 2 | 100.8 |  |
|  | 25619 | MRSA | 2 | 111.3 |  |
|  | 69781 | MSSA | 2 | 138.2 |  |
|  | 36253 | MRSA | 2 | 176.0 |  |
|  | 30462 | MSSA | 2 | 176.9 |  |
|  | 34791 | BORSA or MODSA | 2 | 196.8 |  |
|  | 35994 | MSSA | 2 | 228.1 |  |
|  | 69915 | MRSA | 1 | 273.1 |  |
|  | 13890 | MSSA | 1 | 289.6 |  |
|  | 34427 | MSSA | 2 | 411.8 |  |
| Reference Center for Staphylococci, Brussels, Belgium | 2013S467 | MRSA | 0.064 | 0.4 | Susceptible with  low– relative persister fraction  (S-LP) |
|  | 2014S449 | MSSA | 0.032 | 0.8 |  |
|  | 2017S228 | MRSA | 0.064 | 1.2 |  |
|  | 1078 | MSSA | 0.064 | 1.5 |  |
|  | 2016S354 | MRSA | 0.064 | 1.7 |  |
|  | 1077 | MSSA | 0.064 | 1.7 |  |
|  | 2014S092 | MRSA | 0.032 | 2.2 |  |
|  | 2014S118 | MRSA | 0.064 | 2.4 |  |
|  | 238 | MSSA | 0.064 | 2.9 |  |
|  | 2015S580 | MSSA | 0.064 | 3.0 |  |
|  | 1159 | MSSA | 0.064 | 3.0 |  |
|  | 196 | MRSA | 0.032 | 6.7 |  |

|  | 1214 | MSSA | 0.032 | 26.7 | Susceptible with  high– relative persister fraction  (S-HP) |
| --- | --- | --- | --- | --- | --- |
|  | 2015S418 | MRSA | 1 | 53.6 | Resistant with  high– relative persister fraction (R-HP) |
|  | 2016S605 | MRSA | 2 | 154.8 |  |
|  | 2013S092 | MRSA | 1 | 221.7 |  |
|  | 2017S351 | MRSA | 2 | 250.6 |  |
|  | 2017S017 | MRSA | 2 | 301.4 |  |

^a^ determined based on PCR of *mecA* and *mecC* genes and MIC of cefoxitine

^b^ categorization as susceptible (MIC ≤0.25 mg/L) or resistant (MIC > 0.25 mg/L) was made according to the 2019 interpretive criteria of the European Committee for Antibiotic Susceptibility Testing (https://www.eucast.org).

^c^ ratio between the percentage of persisters after 5 h incubation with moxifloxacin at 100 x MIC for the clinical isolate and for ATCC25923; values are mean of 3 independent experiments performed in triplicates

**Table S2: primers used in this study RT-PCR and PCR experiments**

| **Gene** | **Primer** | **Primer sequence (5’→3’)** | **Purpose** | **Product size (bp)** | **Reference** |
| --- | --- | --- | --- | --- | --- |
| - *gmk^$^* | gmk-F | TCAGGACCATCTGGAGTAGGTAAAG | qRT-PCR | 108 | (Truong-Bolduc et al., 2012) |
|  | gmk-R | TTCACGCATTTGACGTGTTG | qRT-PCR |  |  |
| - *recA* | recA-F | GTAGCGCTTCACGCTATTGCT | qRT-PCR | not disclosed | (Cirz et al., 2007) |
|  | recA-R | TTCAAGACCTTGTTCACCATGATC | qRT-PCR |  |  |
| - *lexA* | lexA-F | GAAACGATTCATGTGCCAGTTATT | qRT-PCR | not disclosed | (Cirz et al., 2007) |
|  | lexA-R | GTCGCTATTATGTGTCGATGTTAA | qRT-PCR |  |  |
| - *uvrB* | uvrB-F | AATATTCCCAGCCTCTAAAGAAGAA | qRT-PCR | not disclosed | (Anderson et al., 2006) |
|  | uvrB-R | CTCATCTCGTAATTCTTTCAATCGT | qRT-PCR |  |  |
| - *umuC* | umuC-F | TGCGAGTGTTTCTTGTATTG | qRT-PCR | not disclosed | (Schroder et al., 2013) |
|  | umuC-R | CCCTGTCTTGATGCCTAA | qRT-PCR |  |  |
| - *gyrA* | RT-gyrA-F | TCGTGAAGGTGACGAAGTTG | qRT-PCR | 101 | This study^a^ |
|  | RT-gyrA-R | CTGGCGTACGTTTACCATAAC | qRT-PCR |  |  |
| - *gyrB* | RT-gyrB-F | GGTGCTGGGCAAATACAAGT | qRT-PCR | 107 |  |
|  | RT-gyrB-R | TCCCACACTAAATGGTGCAA | qRT-PCR |  |  |
| - *parC* | RT-parC-F | GCGTGACGCTAAAGAAAACC | qRT-PCR | 121 |  |
|  | RT-parC-R | TTCACCTTCAAGCGCAACTA | qRT-PCR |  |  |
| - *parE* | RT-parE-F | GACGTGGTATGCCAACAGGT | qRT-PCR | 124 |  |
|  | RT-parE-R | CCGTGAAGACCACCTGAAGT | qRT-PCR |  |  |
| - *norA* | RT-norA-F | GACATTTCACCAAGCCATCAA | qRT-PCR | 102 |  |
|  | RT-norA-R | TGCCATAAATCCACCAATCC | qRT-PCR |  |  |
| - *norB* | RT-norB-F | GCTACACCATCAACAGATACAGCAA | qRT-PCR | 117 |  |
|  | RT-norB-R | ACTCAATGCGACGCCAAA | qRT-PCR |  |  |
| - *norC* | RT-norC-F | TGGGTTGGAGATGGATTTTC | qRT-PCR | 130 |  |
|  | RT-norC-R | ACAATTAGCCCTGCAACGTC | qRT-PCR |  |  |

| - *gyrA* | Seq-gyrA_F | AATGAACAAGGTATGACACC | PCR | 223 | (Schmitz et al., 2002) |
| --- | --- | --- | --- | --- | --- |
|  | Seq-gyrA-R | TACGCGCTTCAGTATAACGC | PCR |  |  |
| - *gyrB* | Seq-gyrB-F | CAGCGTTAGATGTAGCAAGC | PCR | 250 |  |
|  | Seq-gyrB-R | CCGATTCCTGTACCAAATGC | PCR |  |  |
| - *parC* | Seq-parC-F | ACTTGAAGATGTTTTAGGTGAT | PCR | 459 |  |
|  | Seq-parC-R | TTAGGAAATCTTGATGGCAA | PCR |  |  |
| - *parE* | Seq-parE-F | CGATTAAAGCACAACAAGCAAG | PCR | 375 |  |
|  | Seq-parE-R | CATCAGTCATAATAATTACTC | PCR |  |  |
| ^a^ designed using Primer3 Software (<http://bioinfo.ut.ee/primer3-0.4.0/> ), using the published sequences of *grlA* (also referred to as *parC*) and *grlB* also referred to as *parE*)(Yamagishi et al., 1996) and *gyrA* and *gyrB* (Ito et al., 1994) of *S. aureus* RN4220. | | | | | |

**Table S3: Mutations in QRDR domains of selected isolates^a^**

| **Phenotype** | **Isolates** | **MIC** | ***gyrA*** | ***gyrB*** | ***parC*** | ***parE*** |
| --- | --- | --- | --- | --- | --- | --- |
| reference | RN4220 | 0.064 | - | - | - | - |
| S-LP isolates | 30337 (i) | 0.064 | - | - | - | - |
|  | *30337* (f) – 32 days* | *2* |  | *Leu433Ile^b^* | *Ser80Phe*^b,d^ | *Glu422Asp****^c^*** |
|  | 69474 (i) | 0.064 | - | - | - | Glu422Asp |
|  | *69474* (f) – 35 days* | *2* |  | *Ile454Val^b^* | *Ser80Ala^b^* | *Glu422Asp* |
|  | 69687 (i) | 0.25 | - | - | His103Tyr^b,d^ | Glu422Asp |
|  | *69687* (f) -18 days* | *2* | *Ser84Leu*^b,d^ |  | *His103Tyr* | *Glu422Asp* |
| S-HP isolates | 69505 (i) | 0.032 | - | - | - | Glu422Asp |
|  | *69505* (f) – 15 days* | *2* | *Ser84Leu* | *-* | *-* | *Glu422Asp* |
|  | 69783 (i) | 0.032 | - | - | - | - |
|  | *69783* (f)- 16 days* | *2* | *Ser84Leu* | *-* | *-* | *Glu422Asp* |
|  | 69867 (i) | 0.064 | - | - | - | Glu422Asp |
|  | *69867* (f) -19 days* | *2* | *Ser84Leu* | *-* | *-* | *Glu422Asp* |
| R-HP isolates | 30642 | 2 | Ser84Leu |  | Ser80Phe | - |
|  | 69781 | 2 | Ser84Leu | - | Ser80Phe | - |
|  | 30566 | 2 | Ser84Leu | - | Ser80Phe | - |
|  | 34427 | 2 | Ser84Leu | - | Ser80Phe | - |
|  | 13890 | 1 | Ser84Leu | - | Ser80Phe | - |
|  | 35994 | 2 | Ser84Leu | - | Ser80Phe | - |

^a^ only non-silent mutations are shown

^b^  base changes leading to amino acid substitutions: Ser84Leu: TCA🡺TTA; Leu433Ile: TTA🡺ATA; Ile454Val: ATT🡺GTT; Ser80Phe: TCC🡺TTC; Ser80Ala: TCC🡺GCC; His103Tyr: CAT🡺TAT; Glu422Asp: GAA🡺GAT

^c^ mutations described as not associated with resistance (Schmitz et al., 1998)

^d^ mutations described as associated with resistance (Kwak et al., 2013; Morrow et al., 2011; Roychoudhury et al., 2001).

* in-vitro selected mutants (f) from the corresponding parental isolates (i), after exposure to ½ MIC of moxifloxacin until reaching an MIC of 2 mg/L. The number of days of subcultures needed to reach this MIC is indicated after the name of the isolate.

**Table S4**: Key pharmacodynamic parameters of the concentration-effects experiments shown in Figure 4: values for (i) each individual strain (single experiments with triplicate determinations of cfu’s for each extracellular concentration (30 in total) and fitting of one Hill-Langmuir function (with slope factor set to 1) and (ii) all strains within the same phenotypic group analyzed together (240, 150, or 120 data points, respectively) and used for fitting a single Hill-Langmuir function (with slope factor set to 1) *. All data are shown as the mean value and the corresponding 95% confidence interval (asymmetrical range of values containing the true mean with 95% of certainty)

| **S-LP ^a^** | | | **S-HP ^a^** | | | **R-HP ^a^** | | |
| --- | --- | --- | --- | --- | --- | --- | --- | --- |
| **isolate** | **IC_50_ ^b^** | **E_max_ ^c^** | **isolate** | **IC_50_ ^b^** | **E_max_ ^c^** | **isolate** | **IC_50_ ^b^** | **E_max_ ^c^** |
| **2014S118** | 1.11 0.83 to 1.48 | -2.089 -2.39 to -1.80 | **1214** | 0.862 0.58 to 1.31 | -1.12 -1.45 to -0.78 | **2015S418** | 1.01 0.64 to 1.63 | -1.46 -1.86 to -1.07 |
| **2016S354** | 1.44 0.85 to 2.55 | -2.58 -3.10 to -2.04 | **69505** | 1.29 0.73 to 2.34 | -1.54 -1.90 to -1.20 | **2017S017** | 0.73  0.48 to 1.10 | -1.37 -1.73 to -1.03 |
| **2017S228** | 0.80 0.54 to 1.18 | -1.78 -2.11 to -1.45 | **69783** | 1.92 1.14 to 3.37 | -1.84  -2.09 to -1.24 | **20975** | 1.30 0.68 to 2.68 | -1.59 -2.12 to -1.12 |
| **1078** | 1.00 0.620 to 1.66 | -1.59 -2.03 to -1.19 | **69867** | 3.01 1.36 to 7.52 | -1.61 -2.24 to -1.47 | **13890** | 2.68 1.76 to 4.14 | -0.903 -1.19 to -0.630 |
| **30337** | 1.07 0.673 to 1.76 | -2.18 -2.61 to -1.78 | **36606** | 1.36 0.83 to 2.30 | -1.12 -1.47 to -0.780 |  |  |  |
| **69474** | 1.76 0.93 to 3.58 | -2.265 -2.81 to -1.78 |  |  |  |  |  |  |
| **69687** | 2.11 1.45 to 3.09 | -2.47 -2.85 to -2.11 |  |  |  |  |  |  |
| **34467** | 0.833 0.55 to 1.28 | -1.830 -2.22 to -1.46 |  |  |  |  |  |  |
| **all ^d^** | 1.17 0.98 to 1.39 | -2.10 -2.38 to -1.81 | **all ^d^** | 1.35 0.99 to 1.87 | -1.45 -1.84 to -1.06 | **all ^d^** | 1.14 0.856 to 1.53 | -1.33 -1.80 to -0.85 |

**^*^** see the figure associated to this Table for residual QQ plots checking that the distributions of residuals are Gaussian for all sets of data.

**^a^** Phenotype: S-LP: susceptible and low persister; S-HP: susceptible and high persister; R-HP: resistant and high persister (see main text for definition of susceptible/resistant and low persister/high persister phenotypes).

**^b^** moxifloxacin extracellular concentration (multiples of the MIC for each strain) causing a change in cfu’s half-way between E**_min_** and E**_max_** (defined hereunder)

**^c^** E**_max_**: maximal relative efficacy (decrease of intracellular cfus’ from the post-phagocytosis intracellular inoculum for an infinitely large extracellular moxifloxacin concentration; E**_min:_** minimal relative efficacy (increase of intracellular cfu’s from post-phagocytosis intracellular inoculum for an infinitely low moxifloxacin extracellular concentration (both calculated using the Hill-Langmuir function (with slope factor set to 1) fitted to the data).

**^d^** fit of all data from all strains in a given phenotype to a single Hill-Langmuir function (with slope factor set to 1). Comparison between models involving (i) different Hill-Langmuir functions (each fitted to the data of a single strain) vs (ii) a single function fitted to the data of all strains in each phenotype group, using the Akaike’s information criteria (AIC) showed a probability of >99% for the single function model (differences in AIC: - 119.1, -55.6 and -56.49 for the S-LP, S-HP, and R-HP phenotypic groups, respectively when comparing it with the multiple functions model.

**Residual QQ plots** (for assessment of Gaussian distribution * for the least square regression made to fit Hill-Langmuir functions to the data of the concentration-effects experiments illustrated in Figure 4 and for which the values of the IC_50_ and E_max_ parameters are shown in Table S4). Left panels: plots for distinct Hill-Langmuir function fitted to the data of each individual strain (each symbol corresponds to one strain). Right panels: plots for a single Hill-Langmuir function fitted to the data of all strains with a similar phenotype.

* X axis: actual residual; Y axis: data points are very close to the line of identity, shown in red on the graph, consistent with a Gaussian distribution of the data used for the regression analysis.

**Figure S1**

Expression of the genes encoding proteins involved in SOS response **(A)** or fluoroquinolone targets **(B)** in selected isolates of *S. aureus* with susceptible low-persister (S-LP(i), red [30337, 69474, 69687]), susceptible high-persister (S-HP(i), green [69505, 69783, 69867]) phenotypes and in their resistant mutants selected in-vitro and having reached an MIC of 2 mg/L (S-LP(f) and S-HP(f)) after culture with subMIC concentrations of moxifloxacin (Figure 3). Isolates were incubated for 5 h with moxifloxacin at 100 x MIC in the conditions used for performing the persister assay (Figure 1). Each dot represents the mean of triplicates for one isolate, with horizontal lines showing the mean and SD. Data are expressed as –ΔΔCT, with the value of ATCC 25923 at time 0 h used as a reference (horizontal dotted line). The thin horizontal line shows the expression level measured for ATCC 25923 at this time point. Statistical analysis: paired t test comparing S-LP(i) and S-HP(i) to S-LP(f) and S-HP(f), respectively: * : p<0.05; **: p < 0.01

**Figure S2**

Expression of the genes encoding fluoroquinolone efflux pumps in selected isolates of *S. aureus* with susceptible low-persister (S-LP(i), red [30337, 69474, 69687]), susceptible high-persister (S-HP(i), green [69505, 69783, 69867]) or resistant high-persister (R-HP, blue [30462, 69781, 30566, 13890, 35994]) phenotypes as well as in mutants selected in-vitro from S-LP(i) and S-HP(i) isolates and having reached an MIC of 2 mg/L (S-LP(f) and S-HP(f)) after culture with subMIC concentrations of moxifloxacin (Figure 3). Isolates were incubated for 1h with moxifloxacin at 100xMIC in the conditions used for performing the persister assay (Figure 1). Similar results were obtained after 5 h of incubation. Each dot represents the mean of triplicates for one isolate, with horizontal lines showing the mean and SD. Data are expressed as –ΔΔCT, with the value of ATCC 25923 at time 0 h used as a reference (horizontal dotted line). The thin horizontal line in panels B and C shows the expression level measured for ATCC 25923 at 1h. Statistical analysis: The figures at the top of the graphs shows the p value of a t-test comparing the group of isolates with ATCC 25923 at time 0h. p values lower than 0.05 are squared in red. Paired t test comparing S-LP(i) and S-HP(i) to S-LP(f) and S-HP(f) or ANOVA comparing parental isolates from different phenotypic subgroups revealed no statistical differences.

**References to the supplementary material**

Anderson, K. L., Roberts, C., Disz, T., Vonstein, V., Hwang, K., Overbeek, R., Olson, P. D., Projan, S. J., and Dunman, P. M. (2006). Characterization of the Staphylococcus aureus heat shock, cold shock, stringent, and SOS responses and their effects on log-phase mRNA turnover. *J. Bacteriol.* 188, 6739-6756.

Cirz, R. T., Jones, M. B., Gingles, N. A., Minogue, T. D., Jarrahi, B., Peterson, S. N., and Romesberg, F. E. (2007). Complete and SOS-mediated response of Staphylococcus aureus to the antibiotic ciprofloxacin. *J. Bacteriol.* 189, 531-539.

Ito, H., Yoshida, H., Bogaki-Shonai, M., Niga, T., Hattori, H., and Nakamura, S. (1994). Quinolone resistance mutations in the DNA gyrase gyrA and gyrB genes of Staphylococcus aureus. *Antimicrob. Agents Chemother.* 38, 2014-2023.

Kwak, Y. G., Truong-Bolduc, Q. C., Bin Kim, H., Song, K. H., Kim, E. S., and Hooper, D. C. (2013). Association of norB overexpression and fluoroquinolone resistance in clinical isolates of Staphylococcus aureus from Korea. *J. Antimicrob. Chemother.* 68, 2766-2772.

Morrow, B. J., Abbanat, D., Baum, E. Z., Crespo-Carbone, S. M., Davies, T. A., He, W., Shang, W., Queenan, A. M., and Lynch, A. S. (2011). Antistaphylococcal activities of the new fluoroquinolone JNJ-Q2. *Antimicrob. Agents Chemother.* 55, 5512-5521.

Nguyen, T. K., Argudin, M. A., Deplano, A., Pham, N. H., Nguyen, H. A., Tulkens, P. M., Dodemont, M., and Van Bambeke, F. (2020). Antibiotic resistance, biofilm formation, and intracellular survival as possible determinants of persistent or recurrent infections by Staphylococcus aureus in a Vietnamese tertiary hospital. Focus on bacterial response to moxifloxacin. *Microb. Drug Resist.* 26, 537-544.

Roychoudhury, S., Twinem, T. L., Makin, K. M., Nienaber, M. A., Li, C., Morris, T. W., Ledoussal, B., and Catrenich, C. E. (2001). Staphylococcus aureus mutants isolated via exposure to nonfluorinated quinolones: detection of known and unique mutations. *Antimicrob. Agents Chemother.* 45, 3422-3426.

Schmitz, F. J., Hofmann, B., Hansen, B., Scheuring, S., Luckefahr, M., Klootwijk, M., Verhoef, J., Fluit, A., Heinz, H. P., Kohrer, K., and Jones, M. E. (1998). Relationship between ciprofloxacin, ofloxacin, levofloxacin, sparfloxacin and moxifloxacin (BAY 12-8039) MICs and mutations in grlA, grlB, gyrA and gyrB in 116 unrelated clinical isolates of Staphylococcus aureus. *J. Antimicrob. Chemother.* 41, 481-484.

Schmitz, F. J., Boos, M., Mayer, S., Kohrer, K., Scheuring, S., and Fluit, A. C. (2002). In vitro activities of novel des-fluoro(6) quinolone BMS-284756 against mutants of Streptococcus pneumoniae, Streptococcus pyogenes, and Staphylococcus aureus selected with different quinolones. *Antimicrob. Agents Chemother.* 46, 934-935.

Schroder, W., Goerke, C., and Wolz, C. (2013). Opposing effects of aminocoumarins and fluoroquinolones on the SOS response and adaptability in Staphylococcus aureus. *J. Antimicrob. Chemother.* 68, 529-538.

Truong-Bolduc, Q. C., Hsing, L. C., Villet, R., Bolduc, G. R., Estabrooks, Z., Taguezem, G. F., and Hooper, D. C. (2012). Reduced aeration affects the expression of the NorB efflux pump of Staphylococcus aureus by posttranslational modification of MgrA. *J. Bacteriol.* 194, 1823-1834.

Yamagishi, J., Kojima, T., Oyamada, Y., Fujimoto, K., Hattori, H., Nakamura, S., and Inoue, M. (1996). Alterations in the DNA topoisomerase IV grlA gene responsible for quinolone resistance in Staphylococcus aureus. *Antimicrob. Agents Chemother.* 40, 1157-1163.
